# Association mapping and genomic prediction for resistance to sudden death syndrome in early maturing soybean germplasm

**DOI:** 10.1007/s11032-015-0324-3

**Published:** 2015-05-17

**Authors:** Yong Bao, James E. Kurle, Grace Anderson, Nevin D. Young

**Affiliations:** Department of Agronomy and Plant Genetics, University of Minnesota, 411 Borlaug Hall, St. Paul, MN 55108-6026 USA; Department of Plant Pathology, University of Minnesota, 495 Borlaug Hall, St. Paul, MN 55108 USA

**Keywords:** Association mapping, Early maturity, Genomic selection, Soybean, Sudden death syndrome

## Abstract

Sudden death syndrome (SDS), caused by *Fusarium virguliforme*, has spread to northern soybean growing regions in the US causing significant yield losses. The objectives of this study were to identify loci underlying variation in plant responses to SDS through association mapping (AM) and to assess prediction accuracy of genomic selection (GS) in a panel of early maturing soybean germplasm. A set of 282 soybean breeding lines was selected from the University of Minnesota soybean breeding program and then genotyped using a genome-wide panel of 1536 single-nucleotide polymorphism markers. Four resistance traits, root lesion severity (RLS), foliar symptom severity (FSS), root retention (RR), and dry matter reduction (DMR), were evaluated using soil inoculation in the greenhouse. AM identified significant peaks in genomic regions of known SDS resistance quantitative trait loci c*qSDS001*, c*qRfs4*, and SDS11-2. Additionally, two novel loci, one on chromosome 3 and another on chromosome 18, were tentatively identified. A ninefold cross-validation scheme was used to assess the prediction accuracy of GS for SDS resistance. The prediction accuracy of single-trait GS (ST-GS) was 0.64 for RLS, but less than 0.30 for RR, DMR, and FSS. Compared to ST-GS, none of multi-trait GS (MT-GS) models significantly improved the prediction accuracy due to weak correlations between the four traits. This study suggests both AM and GS hold promise for implementation in genetic improvement of SDS resistance in existing soybean breeding programs.

**Electronic supplementary material:**

The online version of this article (doi:10.1007/s11032-015-0324-3) contains supplementary material, which is available to authorized users.

## Introduction

Sudden death syndrome (SDS) of soybean [*Glycine max* (L.) Merr.], caused by *Fusarium virguliforme* (Aoki et al. 2003), is an important disease that continues to spread across northern growing regions in the USA (Bernstein et al. 2007; Chilvers and Brown-Rytlewski 2010; Malvick and Bussey 2008; Navi and Yang 2008; Kurle et al. 2003), causing significant yield losses in infected fields (Wrather and Koenning 2009). Hyphae penetrate soybean roots and eventually colonize the vascular tissue of the plant causing the development of root rot (Jin et al. 1996). Subsequently, phytotoxin FvTox1 is produced by *F. virguliforme* and translocated to plant leaves during reproductive stages, causing diagnostic foliar symptoms such as leaf scorch (Brar et al. 2011; Jin et al. 1996). Both the root rot and leaf scorch lead to yield losses varying from 5 to 80 % in individual soybean fields greatly affected by environmental conditions (Roy et al. 1997).

Crop rotation is generally ineffective in reducing the occurrence and severity of SDS because *F. virguliforme* in the form of chlamydospore or macroconidia can persist in crop residue and soil for many years (Roy et al. 1997). Therefore, SDS management relies heavily on planting resistant or tolerant cultivars complemented by optimal cultural practices. To date, soybean cultivars with partial resistance to SDS have been identified and developed (Hartman et al. 1997; Mueller et al. 2002, 2003; Njiti et al. 2002; Schmidt et al. 1999). However, no highly resistant soybean cultivars adapted to northern growing regions are yet available for soybean growers to use. Consequently, there is an urgent need to develop early maturing soybean cultivars with effective and durable resistance to SDS.

Developing SDS-resistant soybean cultivars has proven difficult mainly due to the complex genetic basis of SDS resistance, the interaction of pathogen and plant with the environment, and imperfect screening methods. Both the pathogen and disease are greatly influenced by environmental factors such as temperature, soil fertility, soil texture, rainfall, and planting date, which makes characterization and evaluation of cultivar performance extremely challenging (de Farias Neto et al. 2006; Gongora-Canul and Leandro 2011a, b; Jin et al. 1996; Sanogo and Yang 2001; Vick et al. 2003). For example, rainfall and temperature in the early season can lead to great variability in evaluation of SDS resistance in soybean genotypes because cool and wet conditions favor the initial infection of soybean roots by *F. virguliforme*, while weather during reproductive stages influences foliar symptom expression. In order to accurately screen for resistance to SDS, extensive field trials of soybean genotypes across multiple locations and years are necessary.

The genetic architecture of (partial) resistance to SDS is complex. A total of 58 QTL have been reported as providing resistance to SDS in bi-parental mapping populations (www.soybase.org, verified June 11, 2014), and only a few of them have been consistent across mapping populations from different genetic backgrounds (Kazi et al. 2008). However, the resistance loci c*qRfs4* on linkage group C2 (chromosome 6), *cqSDS001* on linkage group D2 (chromosome 17), *cqRfs1*, *cqRfs2*, *cqRfs3* on linkage group G (chromosome 18), and *cqRfs6* on linkage group N (chromosome 3) were repeatedly mapped in multiple populations (de Farias Neto et al. 2007; Hnetkovsky et al. 1996; Iqbal et al. 2001; Kassem et al. 2006; Kazi et al. 2008; Lightfoot et al. 2001; Njiti et al. 1998, 2002; Prabhu et al. 1999). Potentially, the genetic markers identified from previous QTL mapping studies can assist in the selection of SDS-resistant cultivars in a timely and resource-efficient manner (Prabhu et al. 1999). Luckew et al. (2013) recently evaluated ten confirmed SDS QTL in F_2_-derived lines from six populations and suggested the possibility of stacking QTL to achieve durable SDS resistance.

QTL mapping in bi-parental populations has been limited by the specific genetic backgrounds of the population under study, which reduces the ability to detect resistance genes. By contrast, association mapping (AM) (Rafalski 2002) provides an opportunity to identify QTL at a higher resolution by taking advantage of historical linkage disequilibrium (LD) in diverse populations. With increasing numbers of single-nucleotide polymorphisms (SNPs) combined with declining costs in genotyping, AM has become an attractive approach for revealing the genetic basis of target traits in crop species (Asoro et al. 2013; Bao et al. 2014; Huang et al. 2010; Mamidi et al. 2011, 2014; Jia et al. 2013; Sukumaran et al. 2012; Zhou and Steffenson 2013; Zhou et al. 2014). A recently published AM study identified QTL for SDS resistance in two soybean panels composed of released cultivars and advanced breeding lines from multiple soybean breeding programs (Wen et al. 2014). Compared to this earlier study, a panel of early maturing soybean lines adapted to short season growing condition was investigated and carefully evaluated below-ground phenotypes including root lesion severity and root retention in the greenhouse. The results suggest that SDS disease analysis based on root symptoms can be more informative with higher heritability than other approaches to SDS phenotyping.

A new marker-based approach known as genomic selection (GS) could potentially be an alternative strategy to stack numerous genes to achieve durable SDS resistance. Rather than utilizing only molecular markers in tight association with targeted QTLs, GS has been developed with the aim of directly predicting genetic value for quantitative traits by taking advantage of all available genome-wide marker information (Bernardo and Yu 2007; Meuwissen and Goddard 2010). In the GS scheme, QTL mapping is replaced by genomic prediction model training which involves fitting both phenotypic and genotypic data from a training population in either linear or nonlinear models. Marker effects estimated from the models are subsequently summed up to estimate genomic breeding values of individuals in a validation or breeding population with only genotypic data. Previous results in crop species, including soybean, have indicated that GS holds the potential to improve disease resistance with complex genetic architecture in breeding programs (Bao et al. 2014; Lorenz et al. 2012; Rutkoski et al. 2012, 2014). Here, we seek to investigate the potential use of GS to select SDS resistance in a typical public soybean breeding program with a focus on early maturing germplasm.

## Materials and methods

### Population, genotyping, population structure, and linkage disequilibrium

Details about the population and genotyping strategy were described previously, as were characterization of the population structure and linkage disequilibrium (LD) (Bao et al. 2014). Briefly, 282 soybean lines were selected including ancestral lines, advanced breeding lines, released public cultivars, and landraces from University of Minnesota Soybean Breeding Program (Table S1; Bao et al. 2014). An Illumina GoldenGate assay with 1536 SNP markers was used to genotype the selected soybean lines (Hyten et al. 2010). A total of 1247 SNP markers with greater than 5 % minor allele frequency (MAF) and missing data rate less than 50 % were used in subsequent analyses (Bao et al. 2014). Both *STRUCTURE* (Pritchard et al. 2000) and principal component analysis (PCA) identified a pattern of three clusters in the population approximately corresponding to three distinct genetic groups (Table S1; Bao et al. 2014). LD was characterized and illustrated using *Haploview4.2* (Barrett et al. 2005).

### Phenotyping and data analysis

In spring 2013, a total of 279 soybean lines (seeds of three lines were unavailable) were evaluated for SDS resistance in the greenhouse using the inoculation procedure of Luckew et al. (2012). An isolate of *F. virguliforme*, Somerset #1A, originating in Minnesota had been maintained on PDA until it was used to inoculate autoclaved sorghum for use in these screening experiments. The sorghum was prepared for inoculation by soaking 1.5 liter quantities overnight in sterilizable spawn bags (Fungi Perfecti LLC, Olympia, WA) followed by autoclaving and cooling. The cooled sorghum was then inoculated with 15 × 5 mm blocks of PDA infested with 2-week-old cultures of the Somerset #1A isolate. Bags were incubated at room temperature with normal fluorescent room lighting for 30 days. The contents of each bag were mixed daily to ensure uniform infestation of the sorghum throughout the bag. At the time of soybean planting, the growth media was inoculated with a 1:20 (volume/volume) ratio of infested sorghum inoculum to media. The uninoculated control treatment contained only growth media. Each entry was planted in a Jumbo Junior (Belden Plastics Co., St. Paul, MN) square pot containing 800 ml of soil. After planting, the pots were placed in the greenhouse, watered to field capacity daily, and maintained at 22 °C with 14 h daylight.

The greenhouse experiment was conducted as six separate plantings because of space limitations. The six plantings were conducted consecutively under the same greenhouse conditions. Each planting consisted of 30–50 soybean lines with five inoculated replications plus one uninoculated replication for each line. Two check cultivars: ‘McCall’ (susceptible) and ‘MN0302′ (resistant) were included in each planting. For each planting, each plant was evaluated for four symptoms or responses associated with SDS by the same experienced evaluator 4 weeks after planting. These observations included: root lesion severity (RLS), foliar symptom severity (FSS), root retention (RR), and dry matter reduction (DMR).

RLS is a measure of the severity of root lesion development caused by *F. virguliforme* infection ranging from 1 (no lesion) to 10 (most severe lesion development): 1 = no lesions visible on taproot, 2 = lesions on 10 % of the taproot, 3 = lesions on 20 % of the taproot, 4 = lesions on 30 % of the taproot, 5 = lesions on 40 % of the taproot, 6 = lesions on 50 % of the taproot, 7 = lesions on 60 % of the taproot, 8 = lesions on 70 % of the taproot, 9 = lesions on 90 to 100 % of the taproot, and 10 = lesions on >90 % of the taproot of the taproot or the taproot is completely missing.

FSS is a rating of the severity of leaf scorch caused by *F. virguliforme* (Bowen and Slaminko 2008, personal communication; Chawla et al. 2013): 1 = no scorch, 2 = slight symptom development, with mottling on leaves, 3 = moderate symptom development with interveinal chlorosis and necrosis, 4 = intermediate symptom development with interveinal chlorosis and necrosis, 5 = severe interveinal chlorosis and necrosis accompanied by cupping, 6 = interveinal chlorosis and necrosis accompanied by cupping with some defoliation, 7 = most leaves displaying necrosis, and 8 = dead plants.

Percentage of root or shoot dry weight change caused by *F. virguliforme* infection was calculated as RR = (root dry weight of inoculated plant)/(root dry weight of uninoculated plant) × 100. DMR = 100 − (shoot dry weight of inoculated plant)/(shoot dry weight of uninoculated plant) × 100.

The ratings of each trait were then fitted into a linear regression model: *y* = *u* + *L* + *ε* within each planting and performed analysis of variation (ANOVA) with the PROC ANOVA in Statistical Analysis System (SAS) Version 9.4 (Cary, NC), where y was one of the four trait ratings of each plant, *u* was the intercept, *L* was the effect of soybean line, and *ε* was the residual. The effect of line × replication was used as the error term to test significance of the effect of line. The phenotypic value of each soybean line was represented as the mean of trait ratings across five replications for each trait and used the phenotypic values for subsequent AM and GS modeling. Scatter plots were constructed based on the pair-wise correlation between the phenotypic values of each pair of traits.

### Association mapping

We performed AM for RLS, FSS, RR, and DMR, respectively, with mixed linear model in the ‘rrBLUP’ package (Endelman 2011) in R (R Development Core Team 2005). The mixed linear model: *y* = *Xα* + *Pβ* + *Kγ* + *ε* was used, where *y* is the vector of phenotypic values, *X* is the vector of SNP marker genotypes, *α* is the coefficient of marker effect being estimated, *P* is the matrix of first three principal components from PCA accounting for the population structure plus the covariate vector of experimental plantings, *β* is the coefficient of principal components and experimental plantings, *K* is the additive relationship matrix estimated based on SNP genotypes accounting for genetic kinship among the individuals, *γ* is the vector of random effects corresponding to genetic kinship, and ε is the vector of random effects corresponding to residuals. The variances of *γ* and *ε* are Var(*γ*) = *2KV*_g_ and Var(*ε*) = *V*_R_, respectively, where *K* is the genetic kinship, *V*_g_ is the genetic variance, and *V*_R_ is the residual variance. False discovery rate (FDR) of 0.05 was used to correct for multiple comparisons in AM using package ‘QVALUE’ in R (R Development Core Team 2005). SNP markers with FDR *q* value <0.05 were defined as significant SNPs associated with SDS resistance. Given the low SNP density on the genotyping panel, significant SNP markers are not expected to be exact locations of causal genes controlling variation of plant response to SDS. In the vicinity of the significant SNPs, previously described SDS resistance QTL was scanned in soybean genome (www.soybase.org). Manhattan plots were created based on the AM results with SNPEVG (Wang et al. 2012).

### Prediction accuracy of genomic selection

To assess prediction accuracy of genomic selection (GS) for SDS resistance, the same set of phenotypic and genotypic data was used in a ninefold cross-validation study. Specifically, 279 soybean lines first were randomly divided into nine subsets. In each fold, eight subsets of lines (248 lines) were used as training sets and the remaining subset (31 lines) was a validation set. In the training set, the marker effects were simultaneously estimated by fitting a statistical model to both phenotypic and genotypic data. The marker effects were then used to predict the genetic values of individuals in the validation set. Prediction accuracy was calculated as the correlation between marker-based prediction and phenotypic values. The cross-validation process was repeated nine times (nine folds), with every subset of soybean lines used exactly once as the validation set.

### Genomic selection model

Since there are four phenotypic traits associated with SDS resistance in the data set, both single-trait genomic selection (ST-GS) model and multi-trait genomic selection (MT-GS) were evaluated, and their accuracies for predicting SDS resistance were compared. For ST-GS, a mixed linear model was constructed to estimate marker effects of phenotypic traits: $y = Xb + Z\alpha + e$, where *y* is the vector (*n* × 1) of phenotypic observations of *n* individuals, *X* is the design matrix (*n* × *r*) for fixed planting effects, *b* is the vector (*r* × 1) of planting effects, *Z* is the design matrix (*n* × *m*) for additive effects of SNP markers, *α* is the vector (*m* × 1) of additive effects of SNP markers, and *e* is the vector (*n* × 1) of residuals. The variances of *α* and *e* are ${\text{Var}}(\alpha ) = I_{m} \sigma_{\alpha }^{2}$ and ${\text{Var}}(e) = I_{n} \sigma_{e}^{2}$, respectively, where *I*_*m*_ is the *m* × *m* identity matrix, $\sigma_{\alpha }^{2}$ is the additive genetic variance for each maker, $\sigma_{e}^{2}$ is the residual variance, and *I*_*n*_ is the *n* × *n* identity matrix. A computationally efficient method, ridge-regression best linear unbiased prediction (RR-BLUP) was employed to solve the mixed model. Previous GS studies suggested slight differences between different genomic prediction algorithms including G-BLUP (which is equivalent to RR-BLUP), Bayesian approaches, and machine learning algorithms (Asoro et al. 2011; Bao et al. 2014; Lorenzana and Bernardo 2009; Lorenz et al. 2012; Rutkoski et al. 2012). The marker effects were simultaneously estimated by solving the mixed model through the restricted maximum likelihood (REML) method implemented in R package ‘rrBLUP’ (R Development Core Team 2005). Variance of additive effects and variance of residual effects were estimated.

MT-GS models were developed by fitting the phenotypic observations of multiple traits (t) simultaneously in a multivariate mixed linear model: $y = (I_{t} \otimes X)b + (I_{t} \otimes Z)\alpha + e$, where *y* is the matrix (*n* × *t*) of phenotypic observations for t traits of n individuals, *I*_*t*_ is the identity matrix (*t* × *t*), *X* is the design matrix (*n* × *r*) for fixed planting effects for each trait, *b* is the matrix (*r* × *t*) of planting effects for t trait, *Z* is the design matrix (*n* × *m*) for additive effects of SNP markers for each trait, *α* is the matrix (*m* × *t*) of additive effects of SNP markers for *t* trait, *e* is the matrix (*n* × *t*) of residuals, and $\otimes$ denotes the Kronecker product. The variances of *α* and *e* are ${\text{Var}}(\alpha ) = G_{0} \otimes A$ and ${\text{Var}}(e) = R_{0} \otimes I_{n}$, respectively, where *G*_0_ is the covariance matrix (*t* × *t*) of additive effects, A is the additive genetic relationship matrix (*n* × *n*), *R*_0_ is the covariance matrix (*t* × *t*) of residuals, and *I*_*n*_ is the identity matrix (*n* × *n*). The marker effects of each trait were simultaneously estimated by solving the mixed model through REML method implemented in R package ‘rrBLUP’ (R Development Core Team 2005). The pair-wise genetic correlation was estimated as $\sigma_{g12} \sqrt {\sigma_{g11} \sigma_{g22} }$, where $\sigma_{g}$ is the genetic variance–covariance matrix for multiple traits. $\sigma_{g}$ was calculated as $\sum\nolimits_{i = 1}^{m} {{\text{Var}}({\text{SNP}}_{i} )\alpha_{i} \alpha_{i}^{T} }$ (Jia and Jannink 2012), where var(SNP_*i*_) is the genotype variance for SNP_*i*_ and *α* is the vector (m × 1) of additive effects for SNP_*i*_. The additive genetic variance and the residual variance were estimated. Ten types of MT-GS models were developed: RLS_FSS model for RLS and FSS; RLS_RR model for RLS and RR; RLS_DMR model for RLS and DMR; FSS_RR model for FSS and RR; FSS_DMR model for FSS and DMR; RR_DMR model for RR and DMR; RLS_FSS_DMR model for RLS, FSS, and DMR; RLS_FSS_RR model for RLS, FSS, and RR; RR_FSS_DMR model for RR, FSS, and DMR; and FT model for all four traits. A notched boxplot was made to compare the prediction performance of MT-GS models to ST-GS models for each trait. The notch marks the 95 % confidence interval for the medians. In the notched boxplot, the medians significantly differ if two boxes’ notches do not overlap.

### Marker number

The effect of marker numbers on GS accuracy was also determined through nine-fold cross-validation by including random samples of 96, 192, 384, and 768 SNPs from the full marker set. Within each fold, the analysis was repeated 100 times to avoid sampling bias for markers. All prediction accuracies were estimated with R package ‘rrBLUP’ (R Development Core Team 2005). A notched boxplot was made to compare the prediction performance of GS models with different subsets of markers for each trait. The notch marks the 95 % confidence interval for the medians. In the notched boxplot, the medians significantly differ if two boxes’ notches do not overlap.

## Results

### Phenotypic data

Analysis of variance (ANOVA) for each of four SDS resistance traits was conducted within each planting. ANOVA showed the effect of soybean genotypes was significant (*p* < 0.05) in all plantings, except for FSS in plantings 4 and 6, and RR and DMR in planting 5 (Table 1). The lack of significance of genotype effect in ANOVA indicated that the effect of replication × genotype contributed a large portion of the trait variation within the planting.

**Table 1:** ANOVA for four SDS resistance traits within each planting

Planting	Source	RLS		FSS		RR		DMR	
		Df	MS	Df	MS	Df	MS	Df	MS
1	Line	50	7.20**	50	11.55**	49	0.68**	49	0.33**
	Error	198	3.82	198	6.28	194	0.36	194	0.20
2	Line	51	7.44***	51	5.66**	51	0.28**	51	0.19***
	Error	184	3.37	184	3.61	184	0.15	184	0.07
3	Line	46	11.78***	46	7.08***	46	0.62**	46	2.38***
	Error	188	4.66	188	3.53	188	0.36	188	0.16
4	Line	50	9.62*	50	6.41	50	1.13**	50	2.98***
	Error	191	7.85	191	5.53	191	0.66	191	1.03
5	Line	49	9.65***	49	10.20**	49	0.15	49	0.09
	Error	177	4.38	177	5.63	176	0.13	177	0.08
6	Line	31	12.73**	31	4.19	31	18.97***	31	15.22***
	Error	111	7.22	111	3.99	111	1.75	111	1.28

*RLS* root lesion severity, *FSS* foliar symptom severity, *RR* root retention, *DMR* dry matter reduction, *Df* degree of freedom, *MS* mean of square

* *P* ≤ 0.05; ** *P* ≤ 0.01; *** *P* ≤ 0.001

Susceptible and resistant check cultivars were set up to provide the means of comparing phenotyping performance in the six plantings. As expected, the susceptible check ‘McCall’ exhibited high RLS scores ranging from 5.5 to 8.8 within plantings with an exception of 2.4 in Planting 3; the resistant check ‘MN0302’ exhibited low RLS scores ranging from 2.2 to 4.6 within plantings with an exception of 6.4 in Planting 1 (Data not shown).

In general, soybean lines showed a wider range of responses to SDS for both RR and DMR than RLS and FSS scores (Supplemental Fig. S1). The phenotypic data density of RLS was more evenly distributed than that of the other three traits (Supplemental Fig. S1). RLS scores ranged from 2.4 to 10 with a total of 49 lines exhibiting scores less severe than the resistant check ‘MN0302’ (Supplemental Fig. S1). FSS scores ranged from 1 to 8 with a total of 81 lines that did not develop any foliar symptoms plus another 43 less severe than ‘MN0302’ (Supplemental Fig. S1). The range observed in RR was 0–1141 % with a total of 69 lines more resistant than ‘MN0302’ (Supplemental Fig. S1). A total of 29 lines did not show any dry matter reduction plus another 64 lines with DMR less severe than ‘MN0302’ (Supplemental Fig. S1). Based on all four traits associated with SDS resistance, 11 soybean lines consistently exhibited symptoms less severe than that of the resistant check ‘MN0302’ and have potential to be used as breeding parents in the SDS resistance improvement program (Supplemental Table S1).

### Pair-wise correlations of traits

**Fig. 1:**
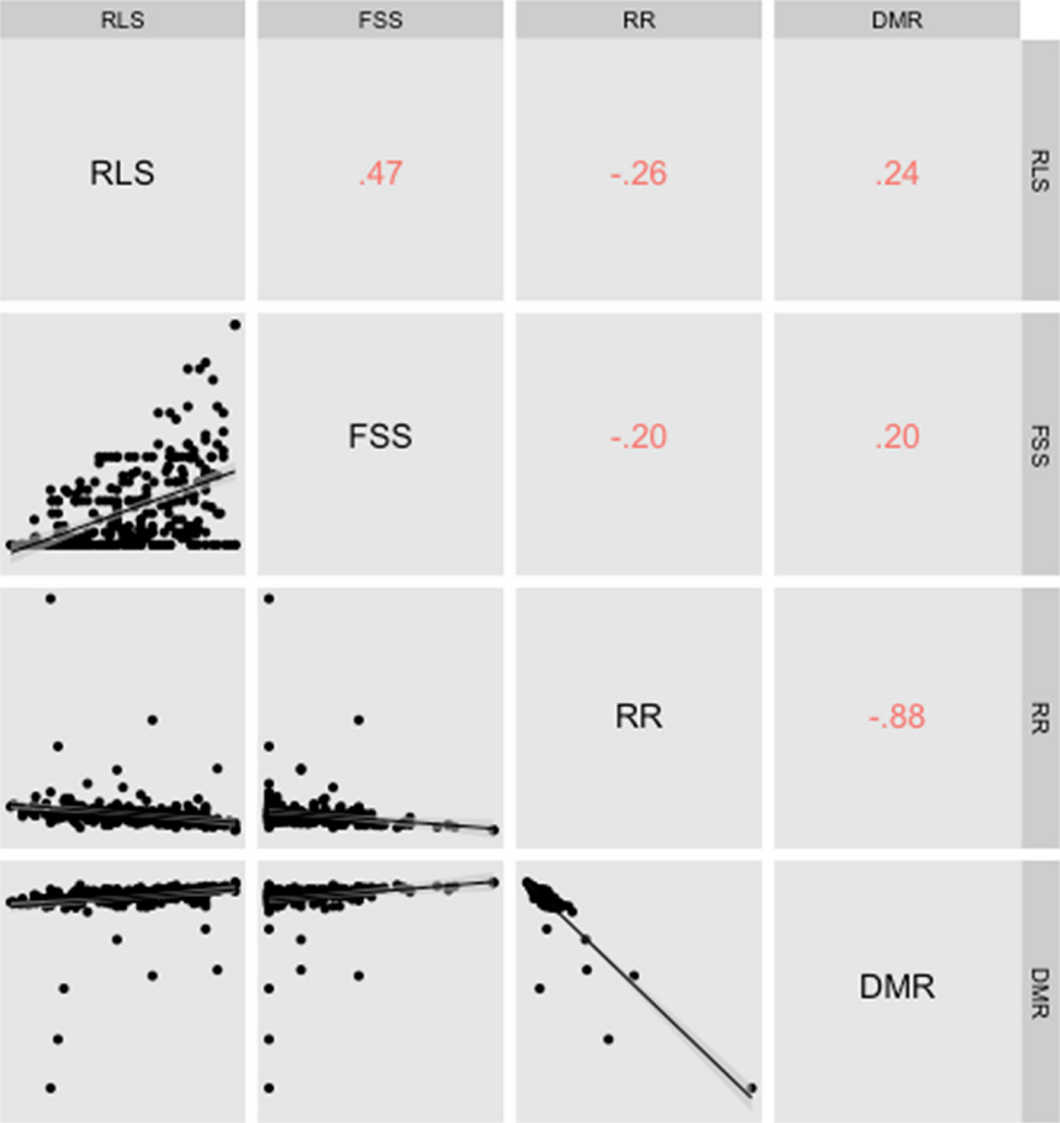
Scatter plots of pair-wise correlation of traits associated with SDS resistance. *RLS* root lesion severity, *FSS* foliar symptom severity, *RR* root retention (%), *DMR* dry matter reduction (%). The values in the scatter plot matrix represent the *r* values of pair-wise correlation of traits

The pair-wise correlations between the phenotypic values of each pair of traits were calculated and are displayed in scatter plots (Fig. 1). As expected, a strong negative correlation was observed between RR and DMR, while RLS and FSS were positively correlated with *r* = 0.47 (Fig. 1). By contrast, the correlations between RR and RLS, DMR and FSS, RLS and FSS, and RR and FLS were poor (Fig. 1). Similar pair-wise phenotypic correlations within each of six plantings (Data not shown) were observed, and pair-wise genetic correlation of traits was consistent with the observation in phenotypic correlation (Supplemental Table S2).

### Significant markers in association mapping analysis

Association mapping (AM) was performed for RLS, FSS, RR, and DMR. The QQ plot indicated the model we implemented for AM was sufficient to control the false positive (Supplemental Fig. S2). We identified two and eight significant (qFDR < 0.05) SNP markers for DMR and RR, respectively, but none for the other two traits (Table 2; Supplemental Fig. S3). Among the eight distinct significant markers, three were in the same genomic intervals as the known SDS resistance quantitative trait loci (QTL) *cqSDS001* on linkage group D2 (chromosome 17) (Table 2; Supplemental Fig. S3). Another marker at position 80.28 cM on linkage group C2 (chromosome 6) was in the genomic region of *cqRfs4* (Table 2; Supplemental Fig. S3). Both *cqSDS001* and *cqRfs4* have been previously identified and confirmed in multiple bi-parental populations (de Farias Neto et al. 2007; Hnetkovsky et al. 1996; Iqbal et al. 2001; Kassem et al. 2012; Kazi et al. 2008; Njiti et al. 2002). Additionally, two significant SNP markers in the present study confirmed a previously identified QTL, *SDS11*-*2*, on linkage group D2 (chromosome 17) (Kazi et al. 2008). The rediscovery of the previously identified QTL strengthened the confidence in the overall quality of AM analysis. Notably, a SNP marker near the telomere on chromosome 3 (linkage group N) was tentatively identified as associated with RR variation, and another SNP marker near the telomere on chromosome 18 (linkage group G) was associated with the variation in both RR and DMR (Table 2; Supplemental Fig. S3; Supplemental Fig. S4). Since no previous QTL had been discovered near these two genomic locations, the two newly identified loci were provisionally named *SDS14*-*1* (on chromosome 3) and *SDS14*-*2* (on chromosome 18). For each of the five loci (namely, *cqSDS001*, *cqRfs4, SDS11*-*2, SDS14*-*1,* and *SDS14*-*2*), RR and DMR peaks were coincident with each other (Supplemental Fig. S4).

**Table 2:** The significant SNPs (false discovery rate < 0.05) detected from association mapping (AM) for SDS resistance

Trait	Marker	LG	Chromosome	Position (cM)	Position (bp)	*P*	qFDR^a^
RR	BARC-044643-08744	N	3	4.71	460,387	2E−04	0.03
	BARC-028177-05786	C2	6	80.28	13,550,856	1E−04	0.02
	BARC-051665-11191	D2	17	72.14	14,849,926	2E−07	0.0002
	BARC-023721-03465	D2	17	75.11	20,352,435	2E−04	0.03
	BARC-064101-18557	D2	17	75.44	25,852,278	2E−05	0.008
	BARC-059487-15840	D2	17	76.12	35,057,016	1E−05	0.006
	BARC-061049-17016	D2	17	77.39	36,090,548	7E−06	0.005
	BARC-024251-04812	G	18	94.3	59,472,567	6E−05	0.002
DMR	BARC-051665-11191	D2	17	72.14	14,849,926	2E−05	0.01
	BARC-024251-04812	G	18	94.3	59,472,567	6E−06	0.008

*RR* root retention, *DMR* dry matter retention, *LG* linkage group

^a^ *qFDR* *q* value of false discovery rate (FDR) estimated with R package “QVALUE.” SNP markers with FDR *q* value < 0.05 were defined as significant SNPs associated with SDS resistance

### Single-trait versus multi-trait genomic selection

**Fig. 2:**
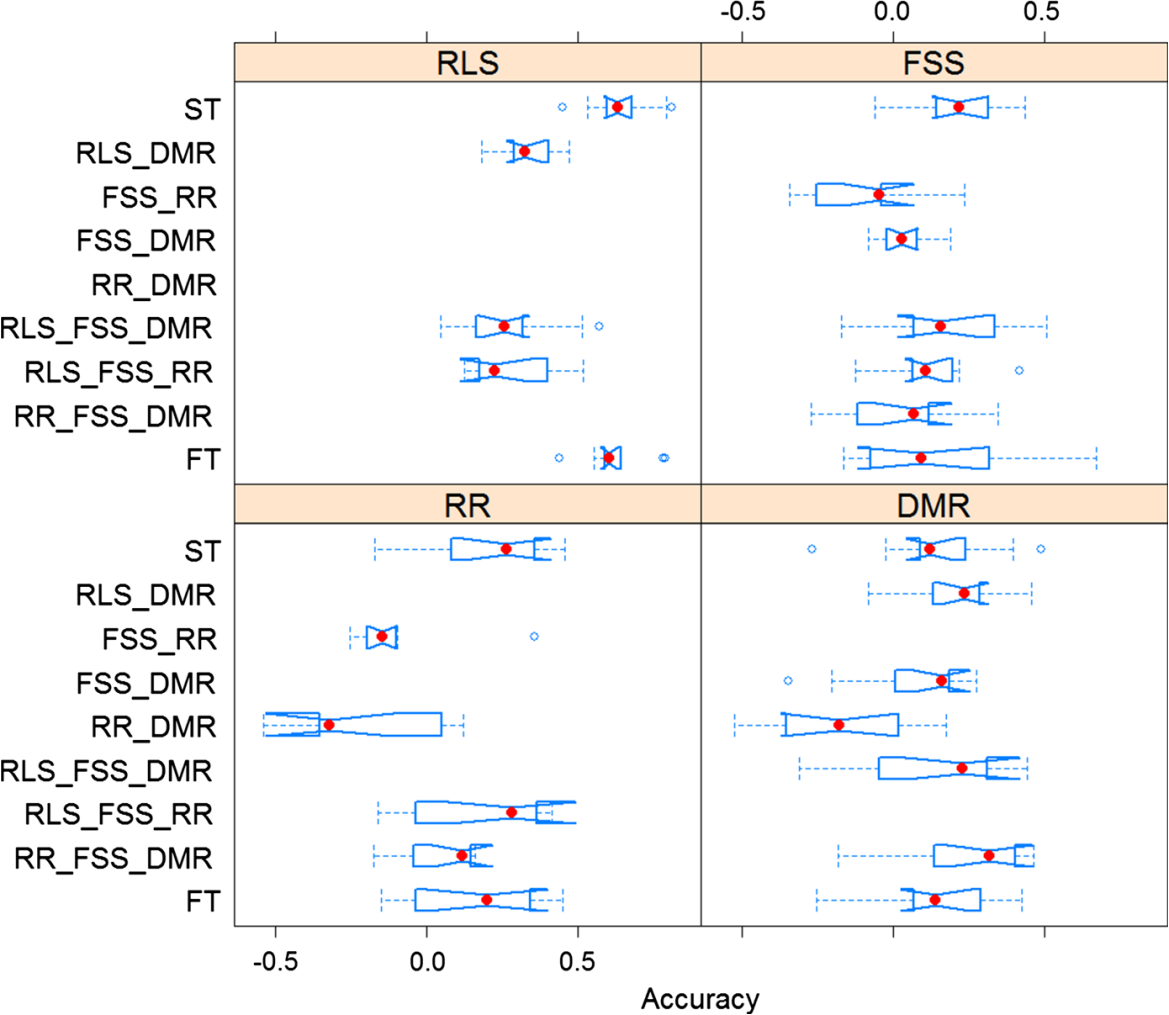
Prediction accuracy with multi-trait genomic selection (GS) models compared with single-trait GS models for four SDS resistance traits. *RLS* root lesion severity, *RR* root retention, *FSS* foliar symptom severity, *DMR* dry matter reduction, *ST* single-trait model, RLS_FSS model for RLS and FSS; RLS_RR model for RLS and RR; RLS_DMR model for RLS and DMR; FSS_RR model for FSS and RR; FSS_DMR model for FSS and DMR; RR_DMR model for RR and DMR; RLS_FSS_DMR model for RLS, FSS, and DMR; RLS_FSS_RR model for RLS, FSS, and RR; RR_FSS_DMR model for RR, FSS, and DMR; and FT model for all four traits. *Red dot* represents median of accuracies for each model. *Notch marks* the 95 % confidence interval for the medians. (Color figure online)

Besides identifying causal loci associated with SDS resistance through AM, the phenotypic and genotypic data sets were used to evaluate the utility of GS in predicting SDS resistance phenotypes. Overall, the prediction accuracy of the ST models was 0.64, 0.20, 0.18, and 0.16 for RLS, FSS, RR, and DMR, respectively (Fig. 2). However, among these ST models, only the prediction accuracy for RLS was significantly different from zero. To determine whether multi-trait (MT) models improved prediction accuracy, ST and MT models were also compared. Compared to ST models, none of MT-GS models significantly improved the prediction accuracy for any trait (Fig. 2). The RLS_FSS_DMR model did increase the prediction accuracy for DMR from 0.16 to 0.25 while maintaining a similar accuracy for FSS, but reduced the accuracy for RLS to 0.26 (Fig. 2). The FT model performed equivalently to ST models for all four traits (Fig. 2).

### Marker number effect on prediction accuracy

**Fig. 3:**
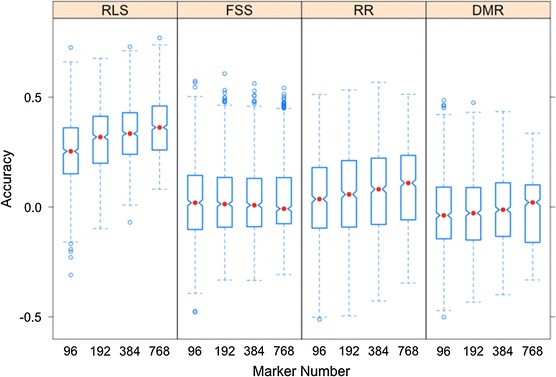
Prediction accuracy with different numbers of markers for four SDS resistance traits. *RLS* root lesion severity, *RR* root retention, *FSS* foliar symptom severity, *DMR* dry matter reduction. *Red dot* represents median of accuracies for each subset of markers. *Notch marks* the 95 % confidence interval for the medians. (Color figure online)

To determine the effect of marker number on prediction accuracy, the prediction accuracy with different sizes of marker set used in ST-GS models was compared. The prediction accuracy generally increased as the number of SNP markers increased for RLS, RR, and DMR, but not for FSS (Fig. 3).The rate of gain in accuracy was greatest when the marker set increased from 96 to 192 (Fig. 3). With 96 random genome-wide SNPs, the prediction accuracy of ST model was only 0.25, 0.02, 0.14, and 0.04 for RLS, FSS, RR, and DMR, respectively (Fig. 3).

## Discussion

Accurate assessment of phenotypic variation is essential for understanding disease biology, effective resistance breeding, and dissection of genetic architecture. The heritability of greenhouse evaluation of SDS resistance ranged from 33 to 66 % in previous studies (Njiti et al. 2001). In the greenhouse experiment, the effect of soybean genotypes was significant (*p* < 0.05) in most plantings indicating an overall reliability of phenotypic data (Table 1). However, we still observed substantial replication × genotype variation in four trait × planting experiments (Table 1). The high level of phenotypic variation between replications has also been observed in previous studies (Kazi et al. 2008; Luckew et al. 2013) and could be attributed to the complex genetic basis of SDS resistance, interactive effects of genotype with environment, and/or imperfect screening methods. Another limitation in the current study was the low throughput capacity of the phenotyping system; soybean lines had to be evaluated in six plantings, which might have reduced the ability to detect all causative QTL and/or led to biased estimations. In other words, the genetic effects might have been confounded by the effect of consecutive experimental plantings conducted over time, limiting the ability to induce SDS symptoms consistently, and as a result, reducing the explanatory power of AM. For example, changing light intensity and ambient temperature variation associated with seasonal changes in sun angle and ambient temperature presumably added to variability in SDS phenotype. To minimize the influences of these sources of variance of among plantings, we conducted the greenhouse experiments with supplemental lighting and air conditioning, and accounted for the effect of plantings as a fixed effect in the AM model.

Eight and two SNP markers in significant association with RR and DMR were identified, respectively, indicating a total of five loci underlying SDS resistance. Among the five loci identified in this study, *cqSDS001* and *cqRfs4* had been previously identified and confirmed in more than one population, which strengthens the confidence of the overall analysis. The *cqSDS001* locus was first discovered at positions 78 and 85 cM on linkage group D2 from the resistant sources PI567374 and Ripley, respectively (de Farias Neto et al. 2007) and was later confirmed in another population derived from Hartwig (Kazi et al. 2008). A second SDS resistance locus, *cqRfs4*, was reported to be associated with foliar resistance (Kazi et al. 2008; Luckew et al. 2013; Triwitayakorn et al. 2005); however, we identified a significant SNP marker, BARC-028177-05786, underlying variation of RR in this QTL interval. Given increasing numbers of SNPs in newly developed genotyping assay for soybean (Song et al. 2013), higher resolution of genetic mapping might pinpoint the potential candidate genes in the genomic regions underlying SDS resistance. Additionally, two SNP markers on linkage group D2 were detected as being significantly associated with RR in our study, which adds support to the SDS11-2 locus identified previously in Kazi et al. (2008).

A cluster of SDS resistance genes, *cqRfs1*, *cqRfs2*, *cqRfs3*, has been repeatedly mapped on linkage group G (chromosome 18) (Chang et al. 1996; Iqbal et al. 2001; Meksem et al. 1999; Njiti et al. 1998, 2002; Prabhu et al. 1999; Kazi et al. 2008), but these genes were not detected in our collection of soybean accessions. More recently, Wen et al. (2014) detected a strong peak at or near the *Rfs2* locus in all the SDS disease assessment criteria evaluated their AM study. Possible explanations for these differences include different sources of germplasm used in different studies as well as the methods used for resistance evaluation. In the present study, the mapping panel germplasm included released cultivars and advanced breeding lines adapted to Minnesota across maturity group 00 to I in contrast to the later maturing germplasm in Wen et al. (2014). In the present study, we also inoculated plants with an isolate of *F. virguliforme* originating in Minnesota. Finally, we extended the detailed examination to target below-ground phenotypes in greenhouse. By contrast, SDS resistance was evaluated in naturally infested fields for above-ground symptoms in Wen et al. (2014). Since both root rot and leaf scorch traits are responsible for yield losses caused by SDS, the loci associated with below-ground phenotypes discovered in the present study should complement to the findings in Wen et al. (2014) and other earlier genetic mapping studies of SDS resistance.

Instead of *Rfs2*, a significant SNP marker BARC-024251-04812 on the opposite end of chromosome 18 was identified, which accounted for variation in both RR and DMR. This SNP was about 1.7 Mb away from a previously described resistance QTL SDS4-2 (Njiti et al. 1998). Another novel locus was tagged by a SNP marker BARC-044643-08744 located near the telomeric region of chromosome 3. These two novel loci could be validated in future investigation of either a bi-parental mapping population or another AM population with a higher density of SNP markers.

To pyramid these resistance QTL into commercial soybean cultivars, the significant SNP markers identified in present study can be developed as a breeder-friendly SNP array for conducting MAS in SDS resistance breeding programs. However, stacking multiple QTL and introgressing them to an adapted elite parent would require considerable resources and time. As an alternative to stacking major SDS resistance genes, GS may provide breeders an opportunity to integrate a broader set of causative loci underlying SDS resistance with the goal of more durable resistant soybean cultivars. Despite successful rediscovery of known QTL for RR and DMR, we failed to identify any significant signals (qFDR < 0.05) for RLS and FSS with AM. This might indicate that the genetic variation of RLS and FSS captured in the population is associated with numerous causative genes each with a small effect. In this case, genome-wide selection as implemented through GS is expected to be more effective than MAS because GS would enable breeders to select candidate lines with higher levels of cumulative resistance to SDS conferred by numerous small effect loci. Estimates of prediction accuracy for RLS were as high as 0.64 (Fig. 2), which is comparable to that for SCN resistance in soybean (Bao et al. 2014), Fusarium head blight resistance in barley and wheat (Lorenz et al. 2012; Rutkoski et al. 2012), and northern leaf blight in corn (Technow et al. 2013). Given the high prediction accuracy, GS holds great potential for implementation in genetic evaluation of breeding candidates in an actual soybean improvement program targeting at SDS resistance.

SDS resistance breeding is further complicated by the existence of two apparently distinct resistance mechanisms involved in expression of root versus foliar responses to SDS (Kazi et al. 2008; Triwitayakorn et al. 2005). Some known QTL confer specific resistance to root rot or foliar scorch, while others confer resistance to both (de Farias Neto et al. 2007; Hnetkovsky et al. 1996; Iqbal et al. 2001; Kassem et al. 2006; Kazi et al. 2008; Njiti et al. 1998, 2002). To develop soybean cultivars with both root and foliar resistance to SDS, multi-trait GS (MT-GS) has the potential to be an effective selection strategy for implementing an SDS resistance improvement program. An MT-GS model is developed by simultaneously fitting phenotypic data from the evaluations of root and foliar symptoms as dependent variables in the model. Subsequently, the MT-GS model using one marker panel leads to simultaneous prediction of both root and foliar symptoms.

Our results suggested that the prediction accuracy of GS model based on single traits (ST-GS) for FSS, RR, and DMR was comparatively low (<0.3) (Fig. 2). In a simulation study, Jia and Jannink (2012) indicated that the prediction accuracy for low-heritability traits could be improved by GS models based on multiple related traits (MT-GS) models. The underlying mechanism of improved accuracy for low-heritability traits in MT-GS is presumably genetic relationship between the highly related traits (Jia and Jannink 2012). In the case of SDS resistance, we hypothesized that MT-GS might be capable of taking advantage of the genetic relationship between low-heritability traits: FSS, RR and DMR, and high-heritability trait: RLS. However, the FT model based on all four SDS resistance traits performed equivalently to the ST models in the study (Fig. 2) and none of the MT-GS models significantly improved the prediction accuracy. An increase in the prediction accuracy for DMR with the RLS_FSS_DMR model was observed, while the RLS_FSS_DMR model failed to maintain similar prediction accuracy for RLS and FSS as that in ST-GS models (Fig. 2).

A simulation study indicated that MT-GS greatly increased the prediction accuracy only when the genetic correlation between two related traits was higher than 0.7 (Jia and Jannink 2012). The MT-GS models performed equivalent to the ST-GS models; this indicates that the genetic basis of FSS, RR, and DMR might not be highly correlated with that of RLS. Indeed, consistently weak pair-wise correlation of FSS × RLS, RR × RLS and DMR × RLS was observed as shown in Fig. 1 and Table S2. Mueller et al. (2002) also suggested that the correlation between root rot and foliar severity was not significant. Considering that root rot is caused by direct infection of *F. virguliforme* (Jin et al. 1996), while foliar scorch is caused by phytotoxin FvTox1 produced by *F. virguliforme* (Brar et al. 2011; Jin et al. 1996), different genetic mechanisms appear to be involved in root versus foliar resistances.

## Conclusion

The present study suggests AM could be used as an alternative method for mapping QTL underlying SDS resistance, and GS holds potential for implementation in genetic evaluation of root lesion severity associated with SDS. We conclude that SDS resistance is a complex disease trait, leading to numerous challenges in evaluating and breeding for SDS-resistant soybean cultivars. Firstly, improving phenotypic screening methods to ensure high-quality and high-throughput evaluation of SDS resistance should remain as an important component of the current SDS breeding program. Secondly, high-density genome-wide markers or sequence-based genotyping methods could be employed to dissect the genetic architecture of SDS resistance more precisely. Lastly, the realized response and cost-effectiveness of GS deserves further investigation in both greenhouse and field prior to implementing GS for developing durable SDS resistance in soybeans.

## Electronic supplementary material

Supplementary material 1 (DOCX 1118 kb)

## Abbreviations

AM: Association mapping
DMR: Dry matter reduction
FDR: False discovery rate
FSS: Foliar symptom severity
GEBV: Genomic estimated breeding value
GS: Genomic selection
LD: Linkage disequilibrium
MAF: Minor allele frequency
MAS: Marker-assisted selection
PCA: Principal component analysis
QTL: Quantitative trait loci
REML: Restricted maximum likelihood
RLS: Root lesion severity
RR: Root retention
RR-BLUP: Ridge-regression best linear unbiased prediction
SDS: Soybean sudden death syndrome
SNP: Single-nucleotide polymorphism
