# Supplementary material for: Association mapping and genomic prediction for resistance to sudden death syndrome in early maturing soybean germplasm
Source: Mol Breed. 2015 May 17;35(6):128. doi: 10.1007/s11032-015-0324-3 (PMC4434860; doi:10.1007/s11032-015-0324-3)
Supplement: Supplementary file 1 — Supplementary material 1 (DOCX 1118 kb) [file 11032_2015_324_MOESM1_ESM.docx]

| **Table S1. A list of 282 soybean accessions in the panel.** | | | | |
| --- | --- | --- | --- | --- |
| **Name** | **Maternal** | **Paternal** | **Year** | **Group** |
| ACME |  | Pagoda | 1991 | C |
| AGASSIZ | SIMPSON | M71-148 | 2000 | B |
| AJMA |  | Flambeau | 1978 | C |
| ALPHA | FAYETTE | MCCALL | 1994 | B |
| ALTONA | PI 194654 | Flambeau | 1991 | C |
| AMSOY | ADAMS | HAROSOY | 2000 | B |
| ANOKA | KOREAN | M42-37 | 2004 | B |
| ARCHER | WILLIAMS 82 + PRX54-59 | BSR101 | 2008 | B |
| BEESON | C1253 | KENT |  | A |
| BELL |  |  |  | B |
| BERT | M74-270 | A78-123018 | 2000 | B |
| BLACK KATO | M70-127 | CENTURY | 2001 | A |
| BURLISON | K74-113-76-486 | CENTURY82-9648 | 1989 | A |
| CAPITAL | Harrow | No.171 | 1931 | B |
| CENTURY L2 | Bonus | Calland | 1991 | A |
| CHICO | 74P.R>13601W | P.R.13612 | 1983 | B |
| CHIPPEWA 64 | CHIPPEWA(8) | BLACKHAWK |  | B |
| CLAY | CAPITAL | RENVILLE | 1991 | B |
| COUNCIL | OZZIE | DAWSON | 1997 | B |
| DAKSOY | SIGCO KG20 | M81-18 | 1997 | B |
| DWIGHT | JACK | A86-303014 | 1998 | B |
| EVANS | MERIT | HAROSOY | 1997 | B |
| FREEBORN | OZZIE | Fayette | 2008 | B |
| GLENWOOD | Peterson 85 | Evans | 1987 | B |
| GRANDE | ANOKA | MAGNA | 2002 | B |
| GRANITE | SIBLEY | BSR 101 | 1992 | B |
| GRANT | LINCOLN | SENECA |  | B |
| HARK | HAWKEYE | HAROSOY |  | B |
| HAWKEYE | MUKDEN | RICHLAND |  | B |
| HODGSON 78 | HODGSON*7 | MERIT | 2002 | B |
| JACK | FAYETTE | HARDIN | 1990 | B |
| JIM | SIGCO KG20 | M81-18 | 1997 | B |
| KASOTA | M73-105 | VICKERY | 2001 | A |
| KATO | M70-127 | CENTURY | 2008 | A |
| LAMBERT | M75-274 | M76-151 | 2002 | B |
| LESLIE | HODGSON 78 | PELLA | 2001 | B |
| M94-246028 | LAMBERT | M92-1651 | 1998 | B |
| M94-275024 | M89-1006 | KATO | 1998 | A |
| M94-278001 | SAPPORO MIDORI | GRANDEM | 2000 | C |
| M94-283002 | TOYOMUSUME | GRANDEM | 1998 | B |
| M95-101005 | AGASSIZ | OT92-8 | 2000 | B |
| M95-116024 | GLACIER | S19-90OLD | 2001 | C |
| M95-118009 | HENDRICKS | S19-90OLD | 1998 | B |
| M95-123006 | PARKER | M92-1631 | 2000 | B |
| M95-123023 | PARKER | M92-1631 | 1998 | B |
| M95-123116 | PARKER | M92-1631 | 2000 | B |
| M95-202018 | M91-297 | AGASSIZ | 2000 | B |
| M95-206027 | M91-94 | ND90-370 | 2000 | B |
| M95-210133 | HARMONY | SURGE | 2000 | B |
| M95-211102 | SD92-1272 | AGASSIZ | 2000 | B |
| M95-215050 | M92-836 | MN1801 | 2001 | B |
| M95-227016 | IA2008R | M91-201 | 2000 | B |
| M95-228092 | M91-557 | ARCHER | 2000 | B |
| M95-261096 | STRIDE | M92-1731 | 2000 | B |
| M95-265118 | IA2008R | LAMBERT | 2000 | B |
| M95-273031 | GRANITE | S19-90 WLD | 2000 | B |
| M95-273035 | GRANITE | S19-90 | 2011 | B |
| M95-274064 | COUNCIL | S19-90 WLD | 2000 | B |
| M95-274114 | COUNCIL | S19-90 WLD | 2000 | B |
| M95-274129 | COUNCIL | S19-90 WLD | 2000 | A |
| M95-274132 | COUNCIL | S19-90 WLD | 2000 | B |
| M95-275008 | M92-1228 | M90-2144 | 2000 | B |
| M95-278001 | M91-419 | M91-471L | 2000 | C |
| M95-278007 | M91-419 | M91-471L | 2000 | C |
| M95-278022 | M91-419 | M91-471 | 2002 | C |
| M95-279015 | M91-464 | M92-996L | 2000 | C |
| M95-279022 | M91-464 | M92-996L | 2000 | C |
| M95-279028 | M91-464 | M92-996L | 2000 | C |
| M95-284082 | M92-1147 | M92-1144E | 2000 | A |
| M95-284113 | M92-1147 | M92-1144E | 2000 | A |
| M95-287060 | TOYOPRO | M92-1144E | 2000 | A |
| M95-287075 | TOYOPRO | M92-1144E | 2000 | A |
| M95-295100 | MINNATTO | PI561.389B | 2000 | C |
| M95-295108 | MINNATTO | PI561.389B | 2000 | C |
| M96-140012 | LAMBERT | LAMB*2/MARCUS 95BC | 2000 | C |
| M96-143031 | FARIBAULT | M92-1591 | 2000 | B |
| M96-356055 | M92-674 | M92-1708 | 2000 | B |
| M96-356062 | M92-674 | M92-1708 | 2000 | B |
| M96-393101 | D-138 | LAMBERT | 2000 | B |
| M96-403029 | MN0901 | M91-113037 | 2000 | B |
| M96-412098 | TOYOPRO | M92-250 | 2000 | A |
| M96-414071 | M92-1216 | KATO | 2000 | A |
| M96-414121 | M92-1216 | KATO | 2000 | A |
| M96-417038 | TOYOPRO | M92-59 | 2000 | A |
| M96-417040 | TOYOPRO | M92-59 | 2000 | A |
| M96-417149 | TOYOPRO | M92-59 | 2000 | A |
| M96-452022 | M92-1149 | M92-836E | 2000 | A |
| M96-452057 | M92-1149 | M92-836E | 2000 | A |
| M96-452059 | M92-1149 | M92-836E | 2000 | A |
| M96-452061 | M92-1149 | M92-836E | 2000 | B |
| M96-473-3-1043 | LAMBERT | F1(M96-714) | 2000 | B |
| M96-521075 | TOYOPRO | TOYOMUSUME | 2000 | B |
| M96-745056 | LAMBERT | F2 M95-306-196 | 2000 | B |
| M97-101025 | ND91-2721 | 9004 | 2001 | B |
| M97-101088 | ND91-2721 | 9004 | 2001 | B |
| M97-115063 | MN0301 | TRACKER | 2001 | A |
| M97-120001 | AGASSIZ | MN0201 | 2001 | A |
| M97-121119 | MN0302 | 9004 | 2001 | B |
| M97-121138 | MN0302 | 9004 | 2001 | B |
| M97-158083 | A95-483031 | M91-947 | 2001 | B |
| M97-159146 | A95-483043 | LAMBERT | 2001 | B |
| M97-164239 | M90-162034 | M91-103023 | 2002 | B |
| M97-201070 | M91-205002 | M92-825 | 2001 | A |
| M97-204114 | M92-836 | M91-198014 | 2001 | A |
| M97-205062 | M91-202001 | M91-151044 | 2001 | A |
| M97-205082 | M91-202001 | M91-151044 | 2001 | B |
| M97-205091 | M91-202001 | M91-151044 | 2001 | A |
| M97-205096 | M91-202001 | M91-151044 | 2001 | A |
| M97-205097 | M91-202001 | M91-151044 | 2001 | A |
| M97-206036 | M92-101016 | M91-151009 | 2002 | A |
| M97-207045 | M91-198043 | M91-151056 | 2002 | A |
| M97-207052 | M91-198043 | M91-151056 | 2002 | A |
| M97-209054 | M93-401031 | M91-198047 | 2001 | A |
| M97-209075 | M93-401031 | M91-198047 | 2001 | A |
| M97-251029 | HENDRICKS | SHIRE | 2001 | B |
| M97-302004 | MN0301 | M96-132-18 GLA*2 | 2001 | B |
| M97-302128 | MN0301 | M96-132-18 GLA*2 | 2001 | B |
| M97-304052 | SURGE | M96-136-20 370*2 | 2001 | B |
| M97-305077 | MN1401 | M96-132-18 GLA*2 | 2001 | A |
| M98-103039 | 40 | M90-144096 | 2001 | B |
| M98-105090 | M92-405 | MN1401 | 2001 | B |
| M98-118006 | LN95-15292 | M92-1631 | 2001 | A |
| M98-134022 | LAMBERT | HARTWIG | 2004 | B |
| M98-210004 | M90-137050 | MN0301 | 2002 | B |
| M98-210060 | M90-137050 | MN0301 | 2002 | B |
| M98-211117 | MN0302 | DAKSOY | 2002 | B |
| M98-234042 | FREEBORN | IA1006 | 2004 | B |
| M98-238010 | LAMBERT | IA1009 | 2004 | B |
| M98-239080 | MN0902CN | IA1008 | 2002 | B |
| M98-239263 | MN0902CN | IA1008 | 2002 | B |
| M98-240104 | M90-178161 | M92-1645 | 2002 | B |
| M98-278072 | M91-198009 | C1937 | 2002 | A |
| M98-279014 | LAMBERT | M91-198014 | 2004 | A |
| M98-283034 | M92-597 | CX1517-53 | 2002 | A |
| M98-283046 | M92-597 | CX1517-53 | 2002 | A |
| M98-308007 | MN1601SP | IA2021E | 2005 | A |
| M98-308016 | MN1601SP | IA2021E | 2002 | A |
| M98-310021 | MN1102SP | M92-1149E | 2002 | A |
| M98-310066 | MN1102SP | M92-1149E | 2002 | A |
| M98-310069 | MN1102SP | M92-1149E | 2005 | A |
| M98-315056 | TOYOSAMARI | TOYOPROE | 2004 | A |
| M98-324017 | M93-135-82 | IA2017E | 2002 | A |
| M98-324060 | M93-135-82 | IA2017E | 2002 | B |
| M98-331009 | MN0302 | M96-133030 | 2002 | B |
| M98-332108 | M91-198009 | M96-133047 | 2004 | A |
| M99-103172 | IA2021 | M96-746-4-2 | 2002 | B |
| M99-113005 | M96-133019 | DWIGHT | 2004 | B |
| M99-113168 | M96-133019 | DWIGHT | 2004 | B |
| M99-118059 | M90-178161 | M92-1645 | 2002 | B |
| M99-121030 | MN0301 | MN1801 | 2002 | B |
| M99-137-1045 | MN1801 | N97-3359-4 | 2002 | B |
| M99-204037 | ND95-1215 | M92-270029 | 2003 | B |
| M99-209070 | TRAILL | MN0302 | 2003 | B |
| M99-215028 | MN0301 | M92-270029 | 2003 | B |
| M99-230063 | IA1006 | MN0302 | 2005 | B |
| M99-246068 | IA2021 | LAN | 2005 | B |
| M99-248011 | RUNO | MN1401 | 2003 | B |
| M99-255012 | LAMBERT | SUI NONG 10 | 2003 | B |
| M99-255036 | LAMBERT | SUI NONG 10 | 2003 | B |
| M99-274166 | PI548.379 | S-1990 | 2003 | C |
| M99-278133 | MN0301 | S-1990 | 2003 | B |
| M99-278137 | MN0301 | S-1990 | 2003 | B |
| M99-278256 | MN0301 | S-19-90 | 2011 | B |
| M99-286047 | IA1008 | 9234 | 2003 | B |
| M99-286050 | IA1008 | P9234 | 2007 | B |
| M99-286148 | IA1008 | 9234 | 2003 | B |
| M99-286149 | IA1008 | 9234 | 2003 | B |
| M99-302003 | M93-137037 | M93-503015E | 2005 | A |
| M99-313054 | M94-283-2-23 | AC ORFORDE | 2005 | B |
| M99-316-1034 | TOYOPRO | M94-283-2-12E | 2005 | A |
| M99-326040 | UM3 | MN1306SPL | 2005 | C |
| M99-327049 | MN0203SP | M94-227084L | 2005 | C |
| M99-329038 | UM3 | PI561.389BL | 2005 | C |
| M99-334034 | M93-399106 | M91-151056LIP | 2005 | A |
| M99-334078 | M93-399106 | M91-151056LIP | 2005 | A |
| M99-337034 | M93-402259 | SURGE | 2005 | A |
| M99-340047 | M90-144096 | KATO | 2005 | A |
| M99-341005 | MN1103SP | MN1004SP | 2005 | B |
| M99-341028 | MN1103SP | MN1004SP | 2005 | B |
| M99-386097 | MN0302 | M96-134044 | 2005 | B |
| MAPLE GLEN | BD22115-13 | PREMIER | 1988 | B |
| MAPLE RIDGE | FISKEBY III | EVANS | 1997 | B |
| MCCALL | (ACME X CHIPPEWA) | HARK | 1997 | B |
| MERIT | BLACKHAWK | CAPITAL |  | B |
| MINNATTO |  |  |  | C |
| MN0081 | MN0302 | 9004 | 2001 | B |
| MN0082SP | MN0202SP | UM3L | 2004 | C |
| MN0091 | GLACIER | S19-90 | 2004 | C |
| MN0092 | ND(M)90-370 | GLACIER | 2001 | B |
| MN0095 | M92-270029 | M93-313185 | 2007 | B |
| MN0101 | M90-137050 | Traill | 2006 | B |
| MN0102SP | MN0202SP | UM3L | 2004 | C |
| MN0103SP | MN0202SP | UM3 | 2010 | C |
| MN0107 | MN0302 | DAKSOY | 2007 | B |
| MN0201 | OZZIE | OT88-11 | 2001 | B |
| MN0203SP | NATTO | M86-2368L SEED | 1998 | C |
| MN0205SP | M91-419 | M91-471L | 2005 | C |
| MN0207SP | UM3 | MN1306SPL | 2005 | C |
| MN0301 | MAPLE DONOVAN | M82-303 | 1997 | B |
| MN0402SP | MN0202SP | UM3L | 2004 | C |
| MN0501SP | MN0303SP | MN1501SPL | 2004 | C |
| MN0502 | MN0302 | PI561.353 | 2002 | B |
| MN0603SP | MN0202SP | UM3L | 2004 | C |
| MN0606CN | MN0901 | MN0902CN | 2007 | B |
| MN0804SP | M91-198036 | KATO | 2005 | A |
| MN0805SP | MINNATTO | NATTOSANL | 2007 | C |
| MN0901 | M83-766 | LESLIE | 1997 | B |
| MN0903SP | TOYOPRO | STURDY | 2002 | A |
| MN0906SP | MN1001SP | MN0203SPL | 2002 | C |
| MN1003SP | KASOTA | Kato | 2008 | A |
| MN1005 | IA2008R | LAMBERT | 2001 | B |
| MN1006CN | LAMBERT | M92-1631 | 2001 | B |
| MN1007SP | MINNATTO | PI561.389B | 2001 | C |
| MN1008SP | M91-202001 | M91-151044 | 2001 | A |
| MN1009 | M91-116124 | MN1301 | 2008 | B |
| MN1010 | M92-836 | ND(M)91-895 | 2000 | B |
| MN1011CN | MN0301 | Dwight | 2006 | B |
| MN1101SP | VINTON 81 | KATO | 2002 | A |
| MN1104SP | C1937 | M91-1876 | 2002 | A |
| MN1105SP | M91-198043 | M91-151056 | 2002 | A |
| MN1106CN | LAMBERT | M94-246152 | 2002 | B |
| MN1202SP | M93-135-82 | IA2017E | 2002 | A |
| MN1203SP | UM3 | IA2024L | 2008 | C |
| MN1305SP | KATO | VINTON 81 | 1998 | A |
| MN1307 | IA1006 | Surge | 2006 | B |
| MN1307SP | M92-1149 | M92-836 | 2001 | B |
| MN1308SP | MN1601SP | IA2021E | 2005 | A |
| MN1406SP | M92-1149 | SIBLEYE | 1998 | B |
| MN1407SP | M92-1147 | M92-1144MEE | 2004 | A |
| MN1409SP | PI592.916 | M93-402312E | 2002 | A |
| MN1410 |  |  |  | B |
| MN1412SP | M93-399106 | M91-151056LIP | 2005 | A |
| MN1503SP | IA2011 | KATO | 1998 | B |
| MN1505SP | M93-402259 | SURGE | 2005 | B |
| MN1603SP | M92-1144 | LS201E | 2005 | A |
| MN1605SP | M90-267 | M92-776L | 2001 | C |
| MN1606SP | M90-764 | M90-2144 SDS | 2011 | B |
| MN1607SP | M90-764 | M90-2144 | 2001 | B |
| MN1802SP | C1453 | SWIFT |  | B |
| MN1804CN | FARIBAULT | IA1006 | 2002 | B |
| MN1805SP | M93-402259 | M93-901096 | 2007 | A |
| MN1806SP | M93-402259 | M93-901096 | 2007 | A |
| NORMAN | ACME | HARDOME | 1997 | A |
| OZZIE | WILKIN | M63-2174 | 1988 | B |
| PARKER | A79-136012 | DAWSON | 2010 | B |
| PELLA | Calland | L66L-137 | 1991 | B |
| PETERSON | Amsoy x PI 248404 | Provar | 1991 | B |
| PI180501 |  |  |  | C |
| PI227565 |  |  |  | C |
| PI257428 |  |  |  | C |
| PI258385 |  |  |  | B |
| PI297503 |  |  |  | C |
| PI297532 |  |  |  | C |
| PI347540C |  |  |  | C |
| PI347550B |  |  |  | C |
| PI372403A |  |  |  | C |
| PI437228 |  |  |  | C |
| PI437267 |  |  |  | C |
| PI437296 |  |  |  | C |
| PI437610A |  |  |  | C |
| PI437994 |  |  |  | C |
| PI438265 |  |  |  | C |
| PI438445 |  |  |  | B |
| PI438454 |  |  |  | C |
| PI445798 |  |  |  | C |
| PI445799 |  |  |  | C |
| PORTAGE | ACME | COMET | 1997 | C |
| PRIDE B216 | CORSOY | WAYNE | 1997 | B |
| PROTO | II-70-504 | II-69-42 | 2004 | A |
| SIBLEY | M68-256 | HODGSON | 2000 | B |
| SIMPSON | STEELE | HODGSON | 1988 | B |
| STRIDE | HACK | LAMBERT | 1997 | B |
| STURDY | M70-127 | CENTURY | 2011 | A |
| SURGE | A86-204022 | KATO | 2011 | A |
| SWIFT | M54-240 | M54-132 | 2008 | B |
| TOYOPRO | M81-610 | M76-349 | 1997 | A |
| TRAILL | M82-996 | A96-492041 | 2011 | B |
| TRAVERSE | Mandarin | Lincoln | 1991 | B |
| UM3 |  |  |  | C |
| VINTON | (HAROSOY X HIGAN) | VINTON*5 | 2001 | B |
| WALSH | ND88-800 | COUNCIL | 2001 | B |
| WEBER | C1453 | SWIFT | 2008 | B |

| **Table S2. Pair-wise genetic correlation of traits associated with SDS resistance.** | | | |
| --- | --- | --- | --- |
|  | FSS^b^ | RR^c^ | DMR^d^ |
| RLS^a^ | 0.51 | -0.19 | 0.16 |
| FSS |  | -0.17 | 0.17 |
| RR |  |  | -0.87 |
| a RLS, root lesion severity. | | | |
| b FSS, foliar symptom severity. | | | |
| c RR, root retention. | | | |
| d DMR, dry matter reduction. | | | |

| **Table S3. Soybean lines exhibiting resistance to SDS for all four traits** | | | | | | |
| --- | --- | --- | --- | --- | --- | --- |
| Name | Maternal^a^ | Paternal^b^ | RLS^c^ | FSS^d^ | RR^e^ | DMR^f^ |
| M99-121030 | MN0301 | MN1801 | 6.8 | 1.0 | 67.1 | 36.4 |
| M99-204037 | ND95-1215 | M92-270029 | 6.0 | 1.0 | 73.4 | 44.5 |
| M99-278133 | MN0301 | S-1990 | 4.0 | 1.0 | 89.4 | 14.8 |
| M99-278256 | MN0301 | S-1990 | 6.5 | 1.0 | 64.0 | 32.9 |
| MN0903SP | TOYOPRO | STURDY | 6.8 | 1.5 | 63.7 | 41.7 |
| MN1008SP | M91-202001 | M91-151044 | 7.4 | 1.0 | 69.3 | 42.9 |
| MN1009 | M91-116124 | MN1301 | 5.2 | 1.4 | 108.0 | 32.1 |
| MN1409SP | PI592916 | M93-402312E | 6.3 | 1.0 | 80.8 | 33.3 |
| MN1503SP | IA2011 | KATO | 5.8 | 1.0 | 63.7 | 47.9 |
| MN1607SP | M90-764 | M90-2144 | 4.6 | 2.6 | 64.9 | 48.9 |
| PROTO | II-70-504 | II-69-42 | 6.0 | 2.4 | 129.3 | -3.5 |
| a Maternal, female parent of soybean line. | | | | | | |
| b Paternal, male parent of soybean line. | | | | | | |
| c RLS, root lesion severity. | | | | | | |
| d FSS, foliar symptom severity. | | | | | | |
| e RR, root retention. | | | | | | |
| f DMR, dry matter reduction. | | | | | | |

| 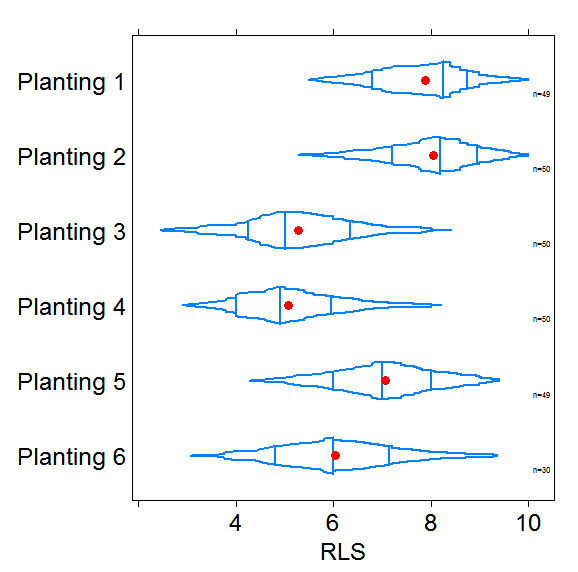 | 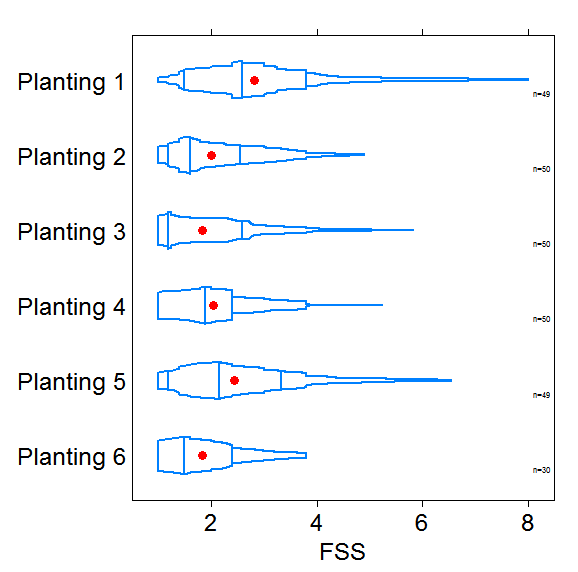 |
| --- | --- |
| 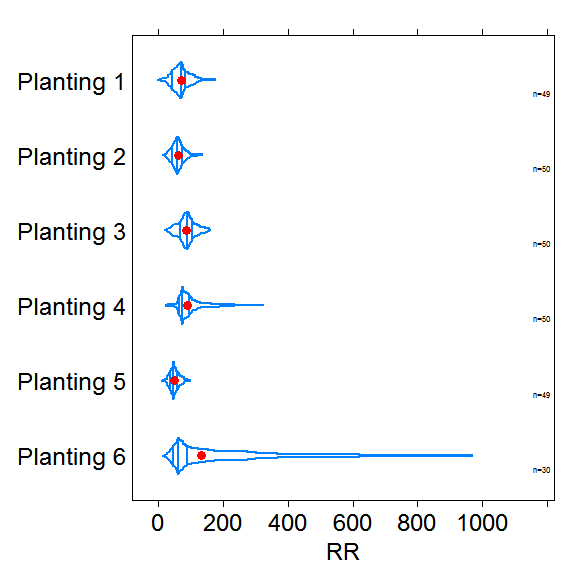 | 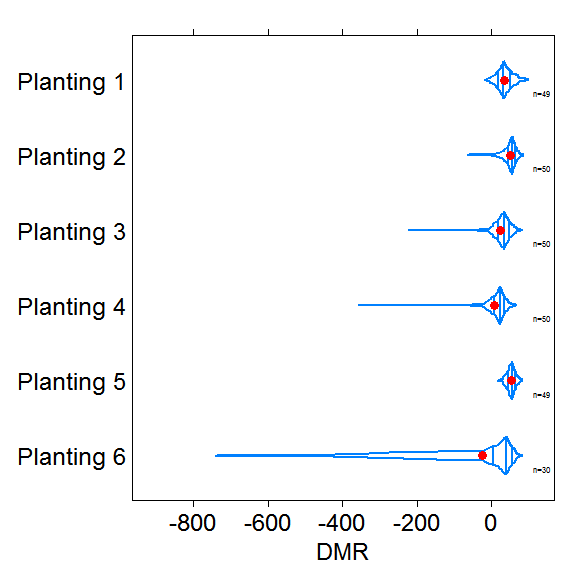 |

**Fig. S1. Box-percentile plots with data density of four SDS resistance traits.** RLS, root lesion severity; FSS, foliar symptom severity; RR, root retention (%); DMR, dry matter reduction (%).

| 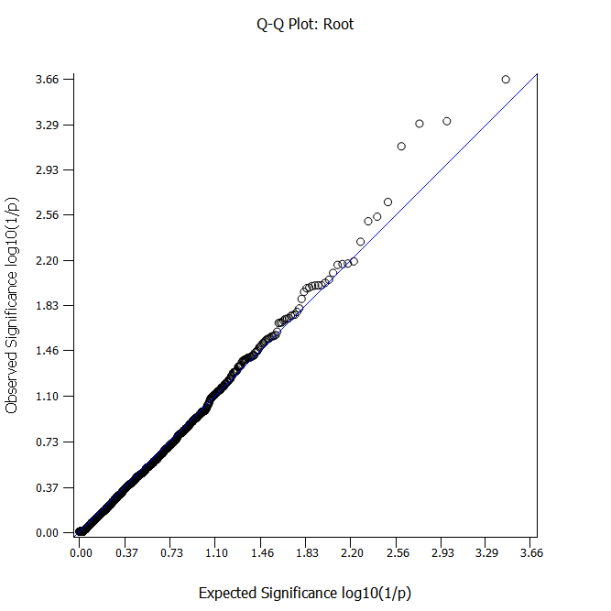  RLS | 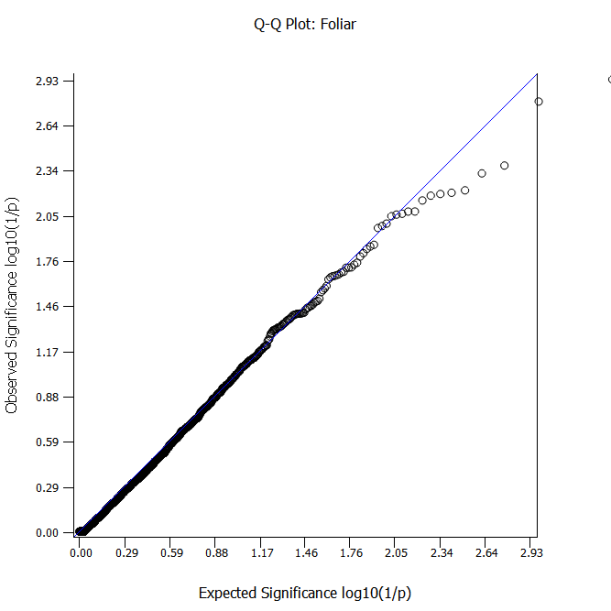  FSS |
| --- | --- |
| 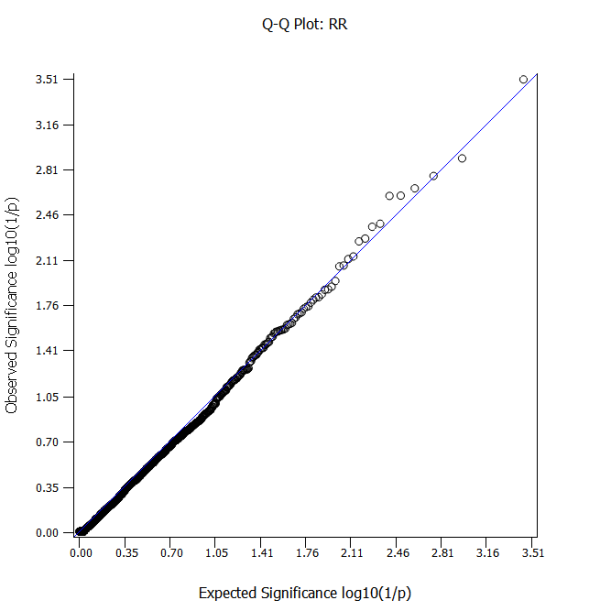  RR | 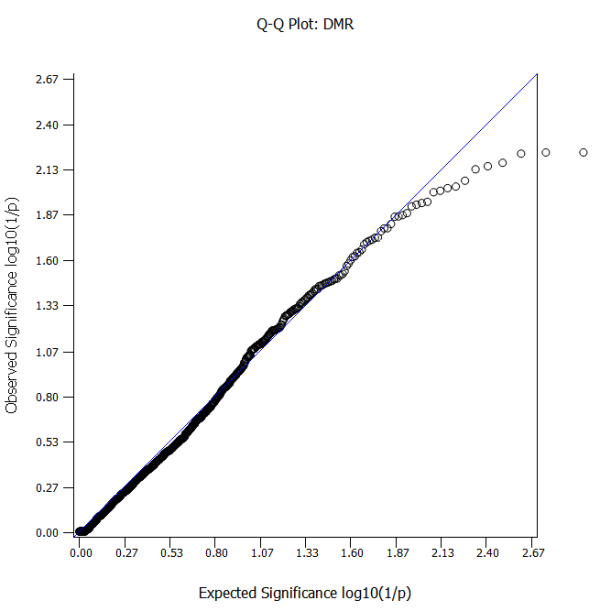  DMR |

**Fig. S2. QQ plots of association mapping of traits associated with SDS resistance.** RLS, root lesion severity; FSS, foliar symptom severity; RR, root retention (%); DMR, dry matter reduction (%).


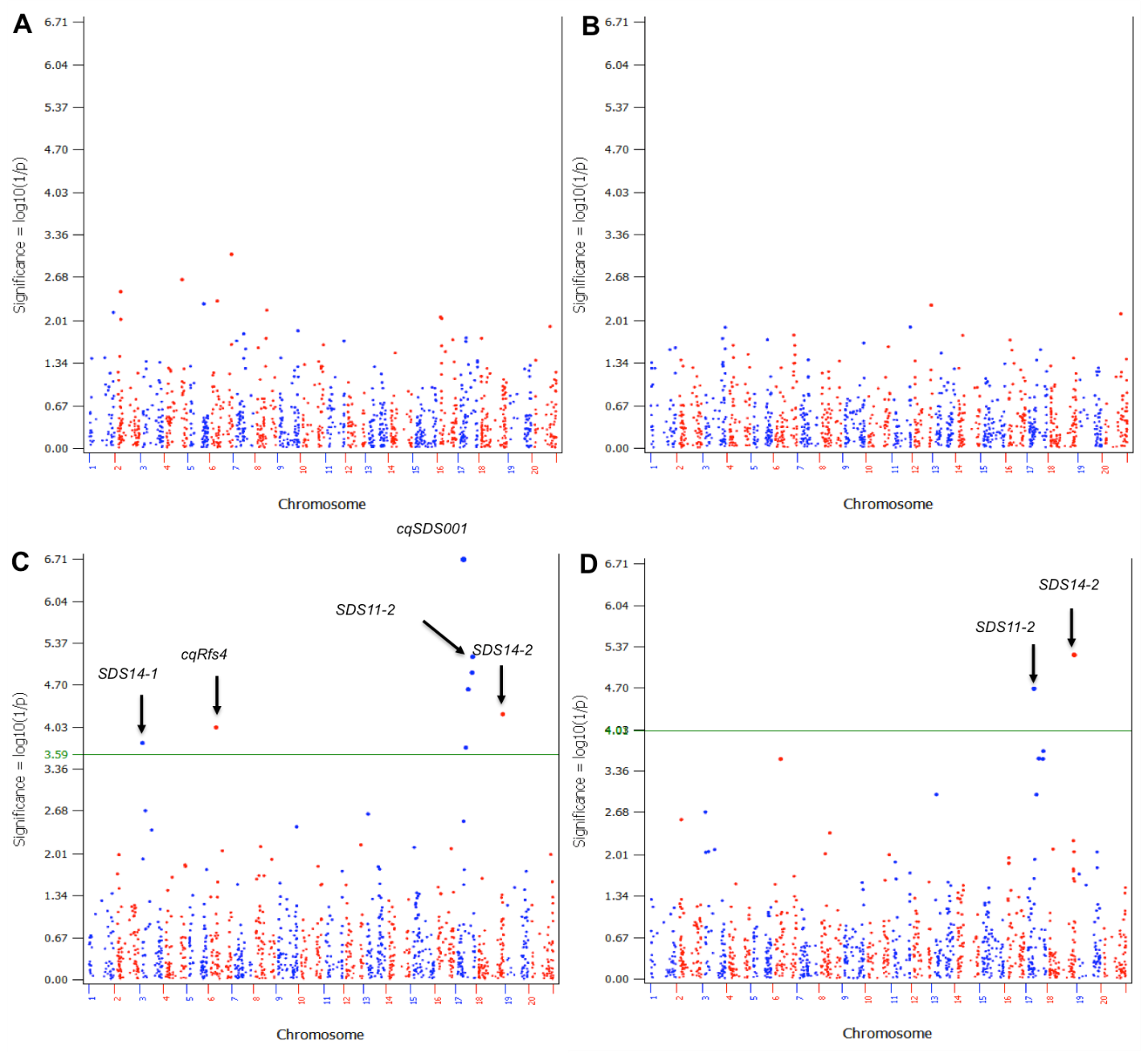


**Fig. S3. Manhattan plots of association mapping for four SDS resistance traits**. A, root lesion severity (RLS); B, foliar symptom severity (FSS); C, root retention (RR); D, dry matter reduction (DMR). The green horizontal line represents the false discovery rate (FDR) of 5%.


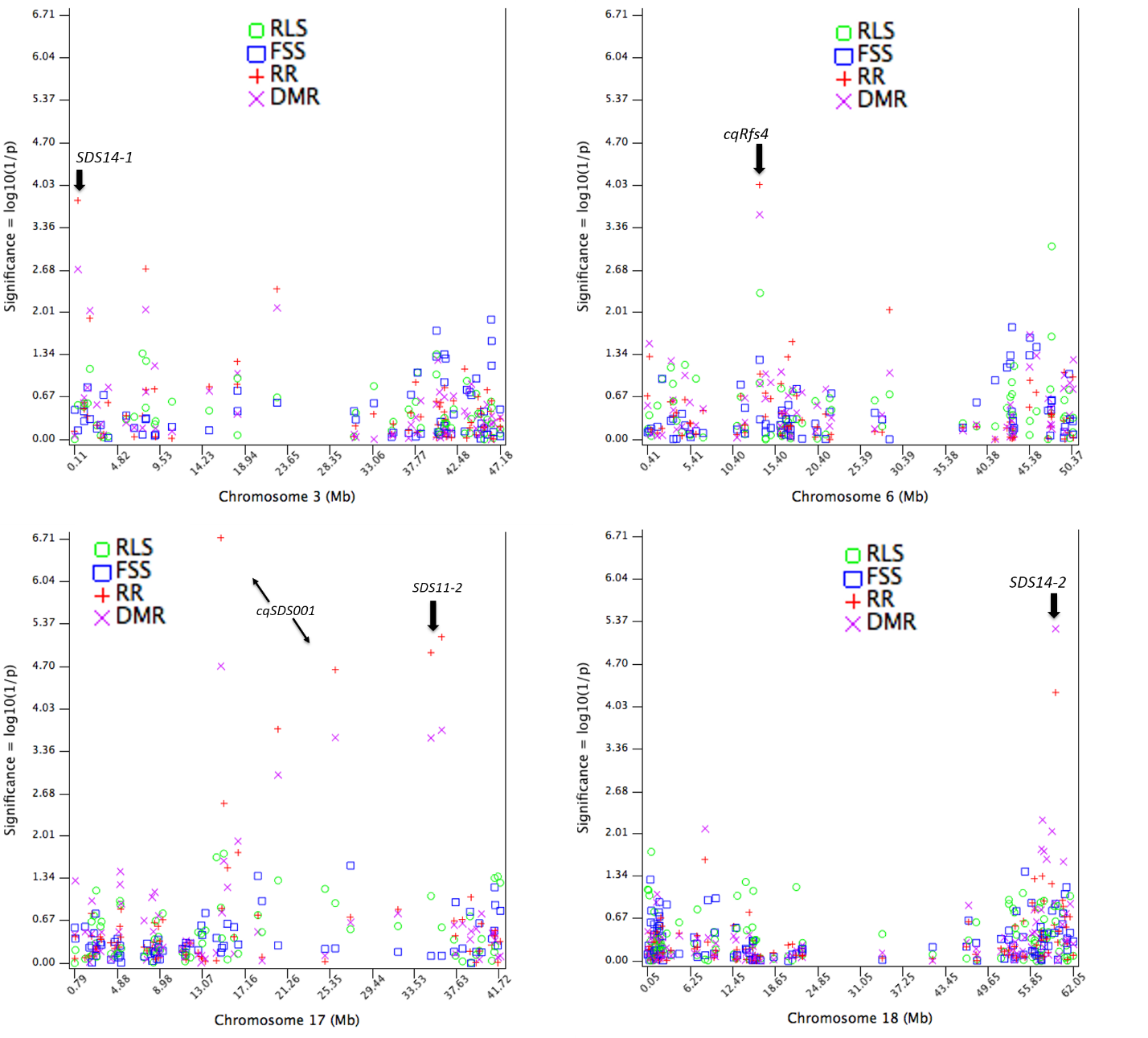


**Fig. S4. Manhattan plots of association mapping for SDS resistance on chromosome 3, 6, 17, and 18**. RLS, root lesion severity; FSS, foliar symptom severity; RR, root retention (%); DMR, dry matter reduction (%).
